# On the Use of Quinine

**Published:** 1847-08

**Authors:** W. S. King

**Affiliations:** U.S.A.


10. *On the use of Quinine, &c.* By W. S. King, M.D., U. S.A. (In a letter to the Editor.)

*Fort Leavenworth*, Feb. 20, 1847.

Dr. C. A. Lee—Sir:—In the New York Jour. Med. for November, 1846, favorable allusion is made to the practice of administering quinine in combination with a diaphoretic. Without being aware that this mode of giving quinine had been recommended or used by others, I have been in the habit of resorting to this combination for some time past, particularly during the past summer.

The novelty and excitement incident to the Santa Fe expedition, caused much anxiety among the sick here, lest their recovery should not be rapid enough to enable them to leave with their companies. The interests of the service also rendered it peculiarly desirable that the cures should be as prompt as possible. As a means of effecting the desirable object, quin. and sp. nitr. dul. (generally, however, with the addition of tinc. opii or tinc. opii comp.) was my usual and favorite prescription. This formula was used, in several hundred cases, of intermittent and remittent fevers, and, in almost every case, with happy effect. With opium, as an adjunct to the quinine and sp. nitr. dul., it will readily be conceived that the value of the prescription is increased, and is rendered capable of fulfilling a much greater number and variety of indications. It may then be employed in those cases of fever, where there is much restlessness and nervous excitement, and, by allaying pain, checking various symptoms, soothing and tranquilizing the patient's feelings, greatly contributes to his comfort and convalesence. The diaphoretic properties of the prescription are much increased by the addition of the opium. The power which this medicine possesses of restoring the equilibrium of the circulation and excitement, by determining to the surface, makes it particularly useful in the hot stages of fever, and enables you to use the quinine sooner than otherwise would be safe.

There are several advantages in this mode of giving quinine. 1st. There is a gain as to time, of twenty-four hours in intermittents, and in remittents, of perhaps several days, which would otherwise be lost in waiting (as recommended by good authority) for the apyrexial intervals, before exhibiting the quinine. 2. By tending to lessen and thereby shorten and speedily terminate the paroxysm of fever, you place the patient, in a short time, in a fair way to recover, and thus diminish the liability to complications which often occur, and in a great measure secure him from the consequences of accidental affections of a distressing and dangerous character. 3. To shorten the paroxysm of fever, and thus destroy the chain of morbid influences that surround the patient *before* strength and restorative powers of the system are weakened, are objects of the highest degree of importance, particularly so in broken down constitutions, or, in such as are feeble and exhausted by previous injurious influence. It needs no argument to show that any means calculated to promote these results must exert an influence highly curative.

I have been much pleased with the success attending this union of remedies, and believing it to be capable of fulfilling the indications I have pointed out, can cordially commend it to those who have not yet tried this method.

Not considering that the use of purgagives or other preparatory treatment was necessary in all cases, I have given the quinine and diaphoretic (with or without the opium according to circumstances) at once on the patient's reporting sick and have had no reason to regret it. By this plan the patient is exempted from the disagreeable and debilitating effects attending the exhibition of emetics and purgatives, which, in many cases may be happily dispensed with. The following formula answers very well in some cases, and where no irritability of the stomach exists, is often preferable to the one already mentioned :

℞.—Tinct. Opii Comp.

Sp. Nitr. Dul. aa. ʒj.

Vin. Antim. gtt. xx.

Solutio Sulphatis Quinæ ʒj.—ʒiij.

Fiat mist. Sumatur alternis horis.

The antimonial was generally omitted in my usual prescription which was similar to the above, with this exception. I do not pretend that in all cases of intermittent and remittent fever, this alone will answer, but that, in experimenting with remedies, or plans of cure, I have found that I could cure and return to duty a greater number of cases, and in a shorter time, by this than by any other medical treatment. I do not offer these considerations because I esteem them new, or worthy of special attention, but for the purpose of bringing the practice of using quinine in combination with various other substances, (which I believe has many advantages over the plan of giving it alone,) to the attention of the profession, with the hope that farther experience will lead to improved modes of exhibiting this article, that will be free from the objections now made by some to the use of this invaluable medicine.—*New York Jour. of Med.*

